# Entrepreneurs Can Know More Than They Can Tell: Conceptualizing and Measuring Tacit Entrepreneurial Knowledge

**DOI:** 10.3389/fpsyg.2022.892223

**Published:** 2022-06-07

**Authors:** Nils Wuytens, Jelle Schepers, Pieter Vandekerkhof, Wim Voordeckers

**Affiliations:** Research Center for Entrepreneurship and Family Firms (RCEF), Faculty of Business Economics, Hasselt University, Hasselt, Belgium

**Keywords:** tacit knowledge, cognition, informal learning, entrepreneurship, qualitative research method, scenarios

## Abstract

Organizational knowledge components dominate research on tacit knowledge. In order to overcome this dominance, we introduce Tacit Entrepreneurial Knowledge (TEK). TEK is conceptualized as one’s experiential learning from past experiences and insights that result in tacit knowledge regarding entrepreneurship that is implicit, personal, and uncodified. For this study the situational judgment test (SJT) approach is adopted to overcome the common limitations in quantifying an individual’s tacit knowledge. The SJT is a scenario-based measurement instrument that allows us to quantify an individual’s TEK. The SJT is developed using three steps: first, scenarios were collected through interviews, followed by formulating responses to the scenarios, and finally, the effectiveness of the responses for each scenario was evaluated. The outcome of this research article is threefold; first, a comprehensive conceptualization of TEK, including delineation of its nomological network. Second, the development of a measurement instrument for TEK and subsequent scoring method. Finally, an antecedent-consequence model which includes potential contingencies associated with these relationships. In the debate on tacit knowledge, our measurement is innovative and relevant, as previous research failed to uncover an individual’s tacit knowledge in the context of entrepreneurship, despite its importance in various entrepreneurial processes. This study aspires to ignite research into TEK by demonstrating important research opportunities unlocked by our conceptualization and subsequent measurement, offering future researchers a wide range of avenues to uncover the black box of tacit knowledge in entrepreneurship.

## Introduction

Tacit knowledge is widely accepted as one of the most valuable resources of any organization (Amit and Schoemaker, 1993). Scholars have provided evidence that tacit knowledge is an essential driver of firm competitiveness (Grant, 1996a; Smith et al., 2005), innovation (Cavusgil et al., 2003; Zhao et al., 2011), and the introduction of new services (Leiponen, 2006). However, entrepreneurship research on tacit knowledge has tended to be dominated by a focus on organizational-level knowledge components such as outsider assistance, firm investments in employees and training, and universities’ geographical proximity (Chrisman, 1999; Le Breton–Miller and Miller, 2006; Ghio et al., 2016). This course of action has yielded considerable advances, such as to how organizations learn (Brown and Duguid, 1991; Huber, 1991), how knowledge is created in organizations (Nelson and Winter, 1982; Nonaka, 1994; Grant, 1996b), and how knowledge affects firm performance (Wiklund and Shepherd, 2003).

This organizational perspective has pushed the research agenda for tacit knowledge in the direction of knowledge transfer (e.g., Kogut and Zander, 1993; Szulanski, 1996; Osterloh and Frey, 2000; Tsai, 2001), without measuring how much, which, or if there is any tacit knowledge whatsoever to be transferred. Yet, the intense focus on organizational-level knowledge components is remarkable as they circumvent the individual-level as the primary source of knowledge within organizations (Nelson and Winter, 1982; Nonaka, 1994; Grant, 1996b).

Indeed, the attunement of tacit knowledge to the individual is especially important in the context of entrepreneurship, as entrepreneurs’ competitive advantages emerge from their idiosyncratic experiences and insights (Hambrick and Mason, 1984; Sternberg, 2004). Entrepreneurs are guided by their own experiences and prior knowledge to respond appropriately to opportunities and leverage resources consistent with their objectives using their entrepreneurial judgments (Shane, 2000; Foss and Klein, 2012; Li et al., 2020). Disregarding the individual has limited our understanding of the individual’s tacit knowledge and understates the entrepreneur’s role at the helm of the venture (Shane, 2000; Shook et al., 2003).

In order to overcome this blind spot, we introduce a new concept: *Tacit Entrepreneurial Knowledge* (TEK). We define TEK as one’s experiential learning from past experiences and insights that result in tacit knowledge regarding entrepreneurship that is implicit, personal, and uncodified. TEK captures an individual’s tacit knowledge as an assemblage of experiences accumulated over a lifetime.

Next, we develop a measurement instrument for TEK. Traditionally, the difficulty in measuring tacit knowledge lies in the elusive nature of the concept. It is challenging to measure tacit knowledge by a standard measurement approach on account of its context-specific, embedded, and subjective character (Gertler, 2003; Tsoukas, 2003). We seek to overcome this important limitation by leveraging an alternative approach to quantify tacit knowledge, namely situational judgment tests (SJT). The SJT is an approach that is much more adapted to measuring and quantifying procedural elements, such as tacit knowledge, than standard approaches (Lievens et al., 2008; Motowidlo et al., 2018). This is because the differentiating factor of the SJTs lies in the use of work-related scenarios, proceeded by a range of potential responses, which is not only a valuable method of exploring entrepreneurship (Shook et al., 2003) but also provides a deeper look into the cognition of the entrepreneur and the social interactions. In addition, the SJT is a well-established selection tool, best known for its predictive power regarding job performance, and has been adapted to fit various industry domains, including academia (Wagner and Sternberg, 1985; Sternberg et al., 1993), accounting (Tan and Libby, 1997; Bol et al., 2018), education (Stemler et al., 2006; Pretz, 2008), management (Colonia-Willner, 1998; Armstrong and Mahmud, 2008). With the introduction of TEK and a subsequent measurement instrument, we make three significant contributions to the field of entrepreneurship.

First, we conceptualize TEK and distinguish the construct from related constructs in its nomological network. A conceptual delineation is vital as it captures the essential characteristics of the construct, its observable manifestations, and the interrelationships (Cronbach and Meehl, 1955). Articulating the nomological network of TEK has important implications for future research and practice, as it enables future researchers to test the empirical validity of TEK in relation to other variables.

Second, by developing a measurement instrument to quantify the TEK of individual entrepreneurs, we address the main empirical challenge of tacit knowledge, which is the operationalization of an individual’s tacit knowledge in the context of entrepreneurship (Sternberg, 2004; Baum et al., 2011; Bird, 2014; Andrews and Smits, 2018). In the debate on tacit knowledge, our measurement instrument is innovative and relevant, as previous research has failed to uncover an individual’s tacit knowledge in the context of entrepreneurship, despite its considerable importance in various entrepreneurial processes (Grant, 1996a; Smith et al., 2005). In addition, our measurement instrument allows for a more in-depth understanding of the entrepreneur’s behavior (Hlady-Rispal et al., 2021) rather than focusing on organizational-level knowledge components, such as technology tacitness and transferability thereof (Kogut and Zander, 1993).

Finally, with both our concept and subsequent instrument development, we aspire to ignite research into TEK. We reveal promising research opportunities by proposing an antecedent-consequence model, including potential contingencies associated with these relationships. The model offers future researchers a wide range of research avenues to uncover the black box of individual-level tacit knowledge in entrepreneurship.

The article is structured as follows. The following section clarifies the need for a new construct for the entrepreneurship domain, with a subsequent conceptualization of TEK. We then present the methodology used to develop the measurement instrument, followed by the validation of the instrument. Lastly, we provide implications for further research, supported by a reflection on our instrument.

## Why a New Concept?

Before developing *Tacit Entrepreneurial Knowledge* (TEK), it is essential to clarify the necessity and value of a new concept to the field. For decades now, tacit knowledge has been scrutinized by many scholars for its elusive nature. The interest has arisen from explorations carried out from a philosophical point of view, such as Ryle (1949), Wittgenstein (1969), Taylor (1989), and Polanyi (2009). Michael Polanyi first described the term tacit knowing in his book, The Tacit Dimension (Polanyi, 2009). Tacit knowing was established on the premise that “we know more than we can tell” (Polanyi, 2009, p. 4) and found its way to various branches of the social sciences, including economics, psychology, and sociology. In the 1980s and 1990s, the focus of organizational studies centered on advancing the theory of the firm, in which knowledge had become an increasingly crucial explanatory factor (Mahoney and Pandian, 1992). The focus on organizational knowledge prompted the research on tacit knowledge and offered insights into how organizations learn (Brown and Duguid, 1991; Huber, 1991), how knowledge is created in organizations (Nelson and Winter, 1982; Nonaka, 1994; Grant, 1996b), how organizations acquire external knowledge (Cohen and Levinthal, 1990), transfer knowledge (Howells, 1996; Szulanski, 1996), both inter- (Tolstoy, 2010; Acs et al., 2013) as intra-organizational (Tsai, 2001; Park et al., 2015), and how knowledge affects firm performance (Wiklund and Shepherd, 2003). A continued insistence toward organizational knowledge is also apparent in entrepreneurship literature addressing tacit knowledge. Most entrepreneurship studies concentrate on an organizational-level component of tacit knowledge, such as outsider assistance for nascent entrepreneurs (Chrisman, 1999), investments in staff and training in the case of family businesses (Le Breton–Miller and Miller, 2006), the proportion of outside researchers and original researchers collaborating in science-based spin-offs (Knockaert et al., 2011). The intense focus on organizational knowledge components is remarkable, considering it bypasses the individual as the primary source of knowledge within organizations (Nelson and Winter, 1982; Nonaka, 1994; Grant, 1996b). Highlighting organizational knowledge components not only eludes the core principle of tacit knowledge, but also devalues the role of the individual entrepreneur at the helm of the firm (Shane, 2000; Shook et al., 2003). For instance, the influence of leading entrepreneurs on their respective venture, as exemplified by Jeff Bezos (Amazon) and Elon Musk (Tesla), cannot be represented by specific organizational components, such as investment in staff and training or the input of outsider assistance, but demands a more direct approach for tacit knowledge at the level of the individual entrepreneur.

Given the importance of tacit knowledge for individuals and organizations, it can be expected that robust measurements or indicators already exist to date—the opposite holds (Robins and Wiersema, 1995; Smith et al., 2009). Tacit knowledge is most often operationalized by proxies. Proxies that focus on the individual tend to overemphasize the importance of experience, such as nascent entrepreneurs’ work experience (Davidsson and Honig, 2003) or entrepreneurial and industrial experience in angel investors (Erzurumlu et al., 2019). In prior research, it has been argued that experience can be misleading and restrictive for tacit knowledge research since experience disregards acquired insights (Levinthal and March, 1993; West and Noel, 2009). In a sense, the proxy experience does not differ from other proxies such as age, certainly not in entrepreneurship. Since it implicitly assumes that tacit knowledge, as measured by experience, deus ex machina will increase and lead to a competitive advantage. So as experience increases, so do the chances of success. Proxies, to some extent, certainly have merit, for instance, in most macro-economic studies (Warhurst and Thompson, 2006). However, to further advance the entrepreneurship field, our ambition should be to uncover more fine-grained research questions and paths, which is not always possible using proxies. For the sake of clarity, this is not a rejection of proxies, but an affirmation for an appropriate measurement instrument for individual-level tacit knowledge, specifically tailored to the domain of entrepreneurship.

## Conceptualizing Tacit Entrepreneurial Knowledge

### Defining the Construct

TEK is defined as experiential learning from past experiences and insights that results in tacit knowledge regarding entrepreneurship that is implicit, personal, and uncodified (Kogut and Zander, 1992; Grant, 1996b). We foresee that individuals with a high level of TEK, based on their own idiosyncratic experiences and insights related to entrepreneurship, will enable a venture to generate competitive advantages (Reed and Defillippi, 1990; Spender and Grant, 1996). It is through the combination of personal experiences that TEK potentially becomes valuable, rare, non-imitable, and non-replaceable, and therefore a crucial resource for both the individual entrepreneur and the entrepreneurial firm (Barney, 1991). TEK thus represents an individual’s experiences, insights, and learning moments regarding entrepreneurship. Therefore, the construct transcends sectors or industries since it emphasizes the individual entrepreneurs’ experiences, not their ventures. Entrepreneurs often recognize opportunities due to the knowledge they previously acquired (Shane, 2000). More specifically, people often have different beliefs based upon an inkling, instinct, or exclusive information, which is critical in discovering entrepreneurial opportunities (Shane and Venkataraman, 2000). This type of information, which can lead to a competitive advantage, is gained through knowledge incurred throughout personal experiences. TEK has a latent presence within any entrepreneur and is instrumental in deploying resources according to their objectives. Entrepreneurs facing identical situations, receiving identical information, and pursuing identical objectives will interpret the situation differently and make different decisions, drawing on their tacit knowledge as entrepreneurs (Foss and Klein, 2012). Nascent entrepreneurs tend to accrue a certain amount of TEK, even with limited entrepreneurial experience. Exposure to entrepreneurial role models, participation in entrepreneurship education, and past work experience in entrepreneurial firms shape each individual’s understanding of entrepreneurship (Peterman and Kennedy, 2003; Zapkau et al., 2017). In sum, as every entrepreneur has a different background (i.e., experience and education), the amount of TEK within the individual entrepreneur can be very heterogeneous since tacit knowledge varies between experts and novices (Wagner and Sternberg, 1985).

Through the conceptualization of TEK as a separate construct, it is clear that if we continue to make progress in this research domain, a measurement instrument for TEK is imperative.

### The Nomological Network for Tacit Entrepreneurial Knowledge

**FIGURE 1:**
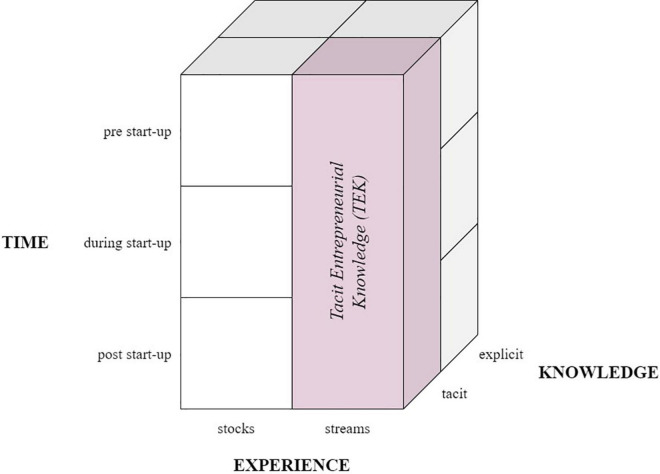
Three-dimensional framework of Tacit Entrepreneurial Knowledge’s (TEK) basic characteristics.

In order to distinguish TEK from other related constructs and position it in the research context, we present a framework based on the essential characteristics of the concept to delineate a nomological network for TEK, in which it occurs (Cronbach and Meehl, 1955). Our framework consists of three dimensions: knowledge, experience, and time (see Figure 1).

The first dimension of the framework relates to the distinction between tacit and explicit knowledge. The distinction between tacit and explicit knowledge offers more insight into how experiences are transformed in an entrepreneur’s memory. Since tacit knowledge is implicitly present in everyone, yet only accessible by “engaging in or running through the skills in which the knowledge is embedded” (Squire, 1986, p. 188). It is a fragment of procedural memory, acquired through personal experience, therefore directly influential for behavior and not readily articulable (Sternberg et al., 2000). In contrast, explicit knowledge is accumulated through formal training or teaching and feeds an individual’s semantic memory. Semantic memory refers to the capacity to store and recollect factual information and is especially necessary for the use of language (Tulving, 1972; Ribas Fernandes and Holroyd, 2017). It is seen as context-free memory concerning facts and is also called the general knowledge about the world (Vargha-Khadem et al., 1997; Squire and Zola, 1998). While TEK is related to Baron and Ensley’s (2006) *cognitive frameworks*, the concepts differ in terms of tacit and explicit knowledge. The cognitive frameworks are also acquired through experience and play a critical role in the recognition of entrepreneurial patterns (Baron and Ensley’s, 2006). But they rely upon a combination of both explicit and tacit knowledge, whereas TEK relies solely on the latter.

The second dimension outlines the perspective of experiences. As Reuber and Fischer (1999) exemplified, experiences can be considered from two perspectives: a continuous stream or a constant stock (Reuber and Fischer, 1999). Since TEK is dynamic and the culmination of event-based experiences to capture an entrepreneur’s ongoing learning process concerning tacit knowledge, TEK can be seen as a continuous stream of experience, upon which past and future experiences are impacted, as opposed to a consistent stock of experience (Reuber and Fischer, 1999; Cope, 2005). In contrast, experiences scrutinized as stocks indicate a steady state at any given time, including depth and breadth of experience, and emphasize an exogenous learning process (Reuber and Fischer, 1999; Westhead et al., 2005). Measures such as age, specific experience (e.g., management, industry, and start-up), and education can be regarded as stocks of knowledge, considering these stocks can be summed together to produce an aggregate.

The final dimension outlines the aspect of time in the TEK construct. Experiential learning in entrepreneurship proceeds in separate dynamic temporal phases (Reuber and Fischer, 1999; Cope, 2005), and research on experiential learning tends to favor experiences and insights that occurred throughout the firm’s lifecycle (Naffziger et al., 1994; Cope, 2005). As opposed to mainstream concepts, TEK is not confined by temporal phases. On the contrary, it considers all insights gained from the entrepreneur’s experiences, whether before, during, or after dissolving the venture. TEK is not limited to a specific temporal phase but instead places the focal point on the life of the individual entrepreneur, even before the start of the entrepreneurial journey. Adjacent constructs, such as *learning-by-doing*, are reduced to learning elements that occur during the firm’s lifecycle (Cope and Watts, 2000).

The previous sections clarified the need for a new construct for the entrepreneurship domain and conceptualized TEK. In the following section, we will present the instrument development process, followed by the validation of the instrument.

## Scenario Development

### Methodology

**FIGURE 2:**
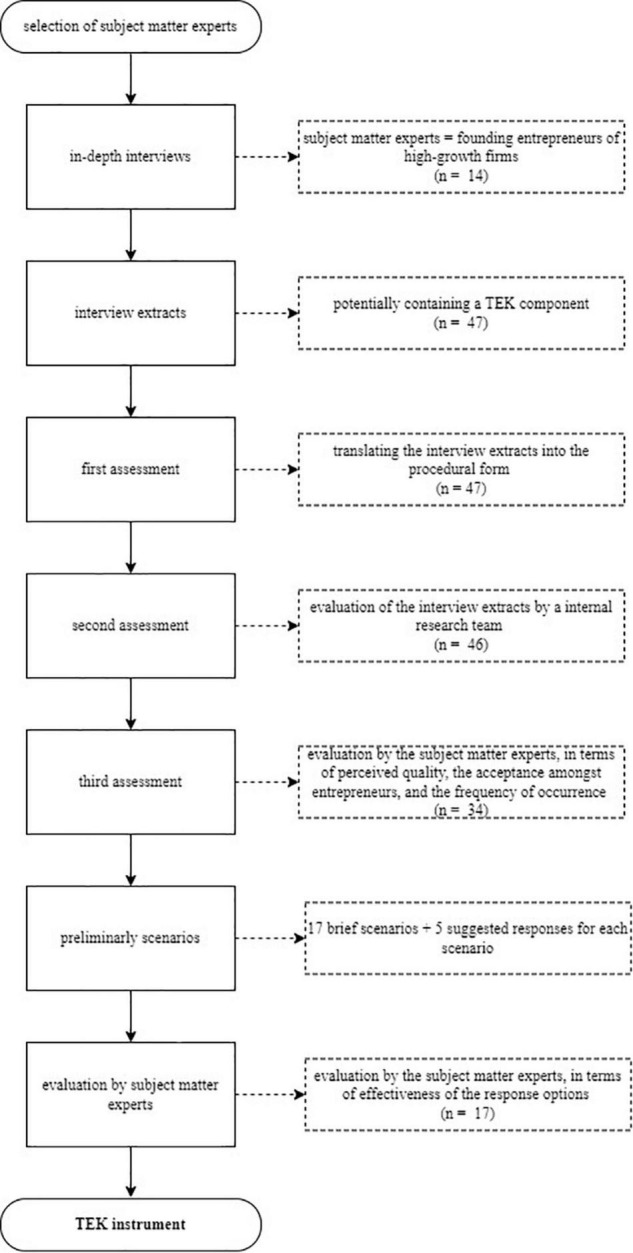
Situational judgment test (SJT) development approach.

Since tacit knowledge is characterized as context-specific, embedded, and subjective, it has for many years been considered extremely difficult to measure with standard measurement methods (Gertler, 2003; Tsoukas, 2003). Therefore, we are turning to an alternative approach, the situational judgment test (SJT). This approach is well-known for quantifying tacit knowledge (Lievens et al., 2008; Motowidlo et al., 2018), because SJTs employ work-related scenarios, followed by a set of possible responses. In so doing, the context is taken into account, and a measurement instrument is created in which reverberations between the entrepreneur’s cognition and social interactions are quantified. For this study, we opted for an SJT developmental approach consistent with the current literature on tacit knowledge (e.g., Wagner and Sternberg, 1985, 1987; Cianciolo et al., 2006; Sternberg, 2006). Developing an SJT is usually characterized by three steps (see Figure 2 for a full description of the development approach): first, the scenarios are collected through interviews, followed by formulating the responses to the scenarios, and finally, the effectiveness of the responses for each scenario is evaluated (Motowidlo et al., 1990; Lievens et al., 2008). As is standard procedure for SJTs, we engage with subject matter experts to develop our instrument. We recruited a panel of 14 subject matter experts based on the following three criteria: first, each entrepreneur needed to be the current and founding CEO of a high-growth firm in order to ensure that the entrepreneur has played a significant role in starting up a business venture (Stuart and Abetti, 1990). The first criterion ensured that the expert entrepreneur had gone through an evolution with the company and excluded inexperienced entrepreneurs from the panel. Second, the venture should apply to the high-growth firm definition, as defined by the European Commission (Eurostat - OECD, 2007), demonstrating an average annualized growth greater than 20% per annum in terms of employees or turnover, over a 3-year period. This criterion is not so much important for the stage in which the organization finds itself (Levie and Lichtenstein, 2010), but its importance lies in the distinguishing capacity of growth-oriented entrepreneurs. Assuming that entrepreneurs have succeeded in growing their new venture into a high-growth firm, we substantiated entrepreneurial success. Finally, there should be no more than two CEOs from corresponding sectors represented in the expert panel. By purposefully eliciting TEK from entrepreneurs active in different sectors, the findings will be allowed to be replicated within divergent sectors (Eisenhardt, 1989).

#### Scenario Development

The subject matter experts were subjected to in-depth interviews following the critical interview technique, which is a qualitative procedure defined by Chell (2004) as “the investigation of significant occurrences (events, incidents, processes, or issues), identified by the respondent, the way they are managed, and the outcomes in terms of perceived effects. The objective is to gain an understanding of the incident from the perspective of the individual, taking into account cognitive, affective and behavioral elements” (p. 48). The mostly unstructured interviews addressed the entrepreneurs’ thought processes and feelings about crucial lessons they had learned over time, and their experience as successful entrepreneurs, emphasizing early career experiences (Sternberg et al., 2000; Taylor et al., 2013). Respondents extensively discussed the essential lessons, described the incidents in detail, and were asked to consider its impact on their development as entrepreneurs and its influence on business development (Chell and Baines, 2000). The entrepreneurs could elaborate on these lessons by clarifying them in the form of cases or stories (Sternberg et al., 2000). All interviews were recorded, and 47 interview extracts of the fragments that potentially contained a TEK component were produced (a detailed description of the extract selection is provided under instrument validity).

#### Responses

The final selection of interview extracts was drafted in brief scenarios, each followed by five suggested responses. The details in both the scenarios and response options were anonymized, such as organizations, entrepreneurs, and industry names, and were constructed using information from the interviews. This process resulted in 17 scenarios based on work-related circumstances, where a problem statement or case was formulated, followed by a range of potential responses specific to the preceding problem or case. Again, the subject matter experts were consulted to evaluate the 17 scenarios and their potential responses on three levels. First, they were asked to assess the consistency between the scenarios and response options or statements. Second, they evaluated the comprehensiveness of the response options, and finally, the quality of the inquiry (Sternberg et al., 2000). Whenever the experts proposed adjustments or indicated inconsistencies, an individual consultation was initiated to discuss the possible adjustments or potential inconsistencies.

#### Evaluation

As a final step in the SJT development approach, the effectiveness of the responses for each scenario was evaluated by the subject matter experts. The subject matter experts were asked to evaluate the final version of the scenario-based measurement. This final consultation round required the members to relay the extent of agreement with each scenario’s subsequent statements, using a seven-point Likert scale to develop the expert mean responses. After all, panelists had given their evaluation ratings, and the TEK instrument was created, consisting of 17 scenarios^1^ (see example scenario in Table 1).

**TABLE 1:** Example scenario with five statements.

**Scenario 7:** You are the founding CEO of a growing start-up. Your next step as CEO is to create your organization’s reward system for the coming years. The difficult part is that your company consists of different departments, including marketing, forecasting, sales, retail, credit, etc.						

Your goal is to build a successful career as an entrepreneur. Considering the situation, to what extent do you agree with the following statements?						

**1**	**2**	**3**	**4**	**5**	**6**	**7**
						
**Strongly disagree**			**Neither agree nor disagree**			**Strongly agree**

a. I would like to install an overarching bonus system based on company-wide objectives. When certain corporate goals are achieved, all employees, regardless of their department, share in the profits.						
b. I firmly believe in an individual reward system. This enables me to quickly identify and reward the best employees.						
c. I want to develop a well-balanced compensation scheme, with both company-wide and personal targets.						
d. I do not believe that employee motivation is stimulated by offering financial bonuses. That is why I opt for a solid fixed salary, without a bonus system.						
e. I mainly focus on increasing overall employee satisfaction through non-financial components, such as employer reputation, challenges at work, job security, and a good work-life balance. I am also committed to improving the overall employee satisfaction.						

### Methodological Implications

The main objective of this study lies in the development of the TEK construct and its measurement instrument. Due to its implicit and unobservable nature, no objective measurements of the criterion variable are available to date. Therefore, we decided to use the rational scoring approach suggested by Lievens et al. (2008) by relying on the judgments of subject matter experts for the TEK instrument. As opposed to an empirical scoring approach typically conducted on a large pilot sample (Lievens et al., 2008), a rational scoring approach is a commonly accepted scoring method for SJTs and consists of deriving the TEK-score for each scenario by comparing the results to a control group (Bergman et al., 2006). Since an SJT is an auspicious tool for overcoming cross-cultural validity issues (Peng et al., 1997), future researchers can relatively easily compile their own expert responses and subsequent benchmark.

The comparison between participants and the control group is performed by calculating the Mahalanobis distance (MD). The MD standardizes the data and adjusts for correlations among variables and is therefore particularly suitable for TEK since it measures similarity between groups using a multidimensional method across cases and variables (Hair et al., 2014). Typically, the MD is used to detect outliers in multivariate data (Penny, 1996). The standard approach pools all data to determine the centroid (i.e., the average discriminant z-score for all group members) and subsequent outliers from that centroid. In this study, we adopt an alternative approach, where the control group’s centroid is constructed, and the respondent’s distance is measured from that centroid. According to this approach, the control group’s centroid is used as the benchmark to calculate the respondents’ scores. This application is burgeoning in several research fields, such as archaeometry, and has merit over the standard approach (Karacic et al., 2016; Overholtzer et al., 2020). The main advantage is that by omitting the respondents from the control group’s centroid, the respondents are prevented from influencing the control group’s characteristics, leading to an unbiased comparison, especially important in smaller samples (Leese and Main, 1994).

The final scores are derived using a series of calculations^2^ based on 17 scenarios (*j* = 17) where each scenario has five potential reactions (*i* = 5) that can each be scored on a seven-point Likert scale. First, the distance between the respondent’s rating for each item (*x*_*ij*_) and the control group $\left(\bar{\mu }\right)$ is calculated.


$${\text{x}}_{\mathrm{ij}}-\bar{\mu }$$

Second, for each scenario, the inverse of the covariance matrix (*C*^−1^) of the control group’s responses is created.


$$C^{-1}$$

Third, the transpose of the distance between the participant’s rating for each item (*x*_*ij*_) and the expert group $\left(\bar{\mu }\right)$ is calculated.


$${\left({\text{x}}_{\mathrm{ij}}-\bar{\mu }\right)}^{T}$$

Fourth, a *D*^2^ per scenario per respondent is calculated by multiplying the three preceding computations.


$${\text{D}}_{\mathrm{ij}}^{2}=\left({\text{x}}_{\mathrm{ij}}-\bar{\mu }\right)\cdot {\text{C}}^{-1}\cdot {\left({\text{x}}_{\mathrm{ij}}-\bar{\mu }\right)}^{T}$$

Fifth, the MD is characterized by a chi-square distribution, and the number of variables per scenario equals the degrees of freedom (i.e., *df* = 5) (Hahs-Vaughn and Lomax, 2020). In order to calculate a TEK-score per scenario, each *D*^2^ is then transformed based on the respective chi-squared value for 5 degrees of freedom at a 0.001 confidence level, which, according to Hair et al. (2014), equals a critical value of 20.515. The transformation involves adjusting any outcome compared to the respective critical value. On the one hand, if the calculated *D*^2^ is greater than the critical value of 20.515, the TEK-score is transformed to zero. Since the MD is typically used for outlier detection, values greater than the critical value are treated as an outlier and are not assigned a score (Hair et al., 2014). On the other hand, if *D*^2^ is smaller than or equal to the critical value of 20.515, the TEK-score is transformed by subtracting the outcome by the critical value.


$$\text{if}\,{\text{D}}_{\mathrm{ij}}^{2}>20.515\,\text{then}\,{\text{TEK}}_{\mathrm{ij}}=0$$

$$\text{if}\,{\text{D}}_{\mathrm{ij}}^{2}\leq 20.515\,\text{then}\,{\text{TEK}}_{\mathrm{ij}}=20.515-{\text{D}}_{\mathrm{ij}}^{2}$$

Finally, to calculate the final TEK-score, the TEK-scores per scenario are added up. To facilitate interpretation, all scores are converted to 100 by multiplying the final scores by 100 and dividing them by 348.755.^3^


$${\text{TEK}}_{\mathrm{total}}=\left({\text{TEK}}_{j=1}+{\text{TEK}}_{j=2}+\ldots +{\text{TEK}}_{j=17}\right)\cdot \frac{100}{348.755}$$

The final two steps in the score calculation process ensure that TEK represents a score ranging between 0 and 100. The closer a respondent’s score approaches 100, the more significant that individual’s TEK is.

## Instrument Validity

Reliability and validity are critical when developing a new measurement to ensure methodological rigor (Crook et al., 2010). In contrast to standard scale development techniques, our scenario development approach aligns closely with the qualitative case study method (Eisenhardt, 1989). Therefore, following the positive tradition, which is a conventional perspective in qualitative research (Aguinis and Solarino, 2019; Grodal et al., 2020; Pratt et al., 2020), the following four criteria were used to establish the rigor of the outcome scenarios: construct validity, internal validity, external validity, and reliability (Lincoln and Guba, 1986; Eisenhardt, 1989; Yin, 2018).

The *construct validity* is ensured when the scenarios offer a correct reflection of the focal construct, i.e., Tacit Entrepreneurial Knowledge (TEK) (Wigren, 2007; Gibbert et al., 2008). TEK, as a focal construct, was elicited by applying different strategies (Yin, 2018). First, by assembling multiple sources of evidence (i.e., subject matter experts). Second, it was possible to document a structured chain of evidence based on a predetermined protocol (i.e., SJT development approach). Third, a step toward construct validity was taken by subjecting the interview extracts to 3 assessments.

Following Sternberg et al. (2000), the first assessment consisted of translating the excerpts into the procedural form to evaluate whether the interview extract matched the procedural form. The procedural form consisted of a set of antecedent conditions (i.e., IF) and consequent actions (i.e., THEN), allowing fewer interpretation possibilities (Sternberg et al., 2000). These statements were combined by taking the connective of the statements (i.e., AND, OR, or ELSE) (see Table 2). After formatting all interview extracts into a procedural form, 1 extract did not fit into the procedural pattern and was subsequently removed.

**TABLE 2:** Combination of the procedural format and contextual stories.

Example of a coded summary	Example of an interview extract
IF you have an idea to start up a new business venture	“Whenever you have an idea to start up a new business venture, it is important to generate quick wins. Therefore, I’ve learned that it is important to talk to as many people as possible about your new venture ideas. Many people are anxious to discuss their business ideas with others because they fear somebody will steal their ideas. I think that the opposite is true. For example, during a neighborhood barbecue, I told one of my neighbors about our business venture, and suddenly, he said that he wanted to help us. It turned out that he had specific skills that were very valuable to our organization. He was a developer and could help us set up specific developers’ classes. He still works for our organization today and is a valuable workforce!”
AND	
IF you are keen on generating quick wins	
THEN explain your idea to many people within your personal and professional network	
BECAUSE this will sometimes lead to valuable collaborations	

The second assessment involved an internal research team evaluating the remaining extracts. 46 encoded summaries and their subsequent transcribed extracts formed the internal review process’s input. This combination enabled the internal panel members to compare and evaluate the procedural format (i.e., the encoded summaries) and the contextual stories (i.e., interview extracts). The internal research team consisted of 3 researchers^4^ to conduct the internal review. All team members were familiar with the concept of tacit knowledge and evaluated each interview extract individually. The evaluation was based on 2 criteria for tacit knowledge: (1) the knowledge on the extracts had to contain information drawn from the entrepreneurs’ own experiences, insights, and individual learnings, and (2) they should not consist of information that is available in academic articles, books, classes, etc. (Sternberg et al., 2000). After the individual evaluation, the internal reviewers discussed the remaining extracts that were not evaluated unanimously and reached a consensus. This first validation process eliminated 12 interview extracts, reducing the preliminary collection of 46 extracts to 34.

The third assessment consisted of quality control by the subject matter experts, who were asked to rate the remaining 34 extracts according to three dimensions, using a five-point Likert scale (Sternberg et al., 2000). The 3 dimensions entailed (1) perceived quality, (2) the acceptance amongst entrepreneurs, and (3) the frequency of occurrence. We created an overall fit index for the remaining extracts based on the entrepreneurs’ responses across these three dimensions. The overall fit index considered the average across the three dimensions of each extract. As a result of averaging across the three dimensions, all dimensions were equally considered instead of giving weight to a particular dimension. Extracts that scored below average were deleted, resulting in a final selection of 17 interview extracts. Finally, construct validity was achieved by involving the entrepreneurs in every aspect of the validation process, such as the initial interviews, assessing the quality of the extracts, and evaluating the final scenarios on consistency and logic, ensuring that the final result inevitably corresponds to the focal construct.

*External validity* indicates whether the findings are generalizable across contexts and domains (Gibbert et al., 2008; Yin, 2018). The first step toward external validity was taken when refining the boundary criteria for the domain experts. In our research, we sought to interview entrepreneurs from various branches to increase the representativeness of the elicited knowledge. By purposefully including entrepreneurs active in different sectors, the findings will be allowed to be replicated within divergent sectors (Eisenhardt, 1989). The expert panel was selected for its expected relevance in illuminating the concept of TEK and is not meant to be representative of any population. They are selected through theoretical sampling, which Eisenhardt and Graebner (2007) describe as follows: “just as laboratory experiments are not randomly sampled from a population of experiments, but rather, chosen for the likelihood that they will offer theoretical insight, so too are cases sampled for theoretical reasons, such as the revelation of an unusual phenomenon, replication of findings from other cases, contrary replication, elimination of alternative explanations, and elaboration of the emergent theory” (p. 27).

A study’s *reliabilit*y demonstrates the study’s replicability in terms of consistency, logic, and traceability (Lincoln and Guba, 1986; Wigren, 2007; Yin, 2018). This study’s methodology and subsequent scenario development follow a well-documented protocol and approach. Continuous involvement of the expert panel throughout the process, an internal and external evaluation of the interview extracts concerning theory (i.e., internal assessment) and practice (i.e., external assessments), and the precise notation of each step ensure that this study is replicable in other contexts. Replication does not imply, as in quantitative research, that identical findings can be reproduced using the same data and the same procedures. For qualitative research, this is not appropriate or attainable (Pratt et al., 2020).

It is important to note that the preceding four quality criteria are not different criteria but interrelated (McGrath, 1981; Gibbert et al., 2008). An example of this interrelatedness is interaction with the expert panel in relation to the interview extracts. According to Sternberg et al. (2000), the subject matter experts’ quality control results in interview extracts represent experience and indicate entrepreneurial success, elevating the outcome scenarios internal, external, reliability, and construct validity. The construction of a clear scenario development approach also contributed to the internal validity and the procedure’s reliability.

## Discussion and Contributions

Prior research has mainly focused on organizational-level tacit knowledge. Although these studies have provided many valuable insights, the central tenet of tacit knowledge, which is the individual as the primary source of knowledge within the firm, is most often circumvented or neglected. Therefore, this study aimed to develop an individual tacit knowledge measurement for the entrepreneurship domain, namely *Tacit Entrepreneurial Knowledge* (TEK). We believe this is an essential step for entrepreneurship research as, until today, entrepreneurship scholars had to rely on proxies, such as prior experience, to quantify an entrepreneur’s individual-level tacit knowledge.

Throughout this study, valuable lessons from experienced entrepreneurs were elicited, transformed into scenarios, and validated according to established criteria. This qualitative development of a scenario-based measurement for TEK draws on Wagner and Sternberg (1985) groundbreaking work and is based upon a sound methodological foundation (Armstrong and Mahmud, 2008). The sheer breadth and depth of scientific work provided by Wagner, Sternberg, and colleagues on tacit knowledge provide compelling evidence to broaden this method to the field of entrepreneurship.

**FIGURE 3:**
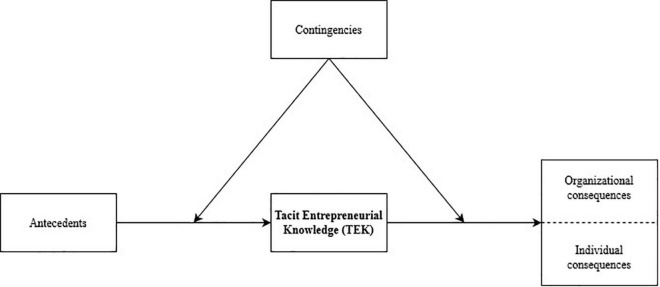
Antecedent-consequence model of Tacit Entrepreneurial Knowledge (TEK).

The introduction of TEK is important to entrepreneurship, as it represents an undebated yet crucial element within the scholarly field. The development of the scenario-based measurement offers the entrepreneurship field the possibility to investigate an entrepreneur’s TEK. Placing TEK at the center of an antecedent-consequence model (see Figure 3) and exploring potential contingencies that serve both ends of these relationships reveals the applicability of this study. Moreover, the contribution of TEK as a construct becomes abundantly clear from an empirical point of view when considering the predictive power of tacit knowledge, which has already been established in other domains (Sternberg, 1997). Such an instrument adds value to the field of entrepreneurship as it enables us to measure a crucial entrepreneurial concept that researchers have failed to grasp so far. It brings to light future research paths that may enrich entrepreneurship literature and advance our understanding of the black box of an individual’s tacit knowledge in entrepreneurship.

### Antecedents of Tacit Entrepreneurial Knowledge

TEK is accumulated as a result of past experiences, insights, and learnings regarding entrepreneurship, which makes it self-evident that TEK is influenced by factors that enable the individual to develop a specific image of entrepreneurship, identify opportunities, and allocate resources to achieve their entrepreneurial objectives. As possible antecedents of TEK, the primary focus in this study lies in an individual’s learning path and how it might be instrumental in the accumulation of TEK.

This ability is reflected in a person’s learning orientation, which is the tendency to cultivate competence by obtaining new skills and comprehending new situations (Dweck, 1986; VandeWalle, 1997; De Clercq et al., 2013). Empirical evidence demonstrates that learning-oriented individuals are consistently open to new experiences (VandeWalle et al., 2001), prefer to engage in more challenging tasks (Elliott and Dweck, 1988), and appreciate activities that might enhance personal development (VandeWalle and Cummings, 1997). As it is a fact that tacit knowledge is best transmitted through social interactions and experiences (Cook and Brown, 1999; Haldin-Herrgard, 2000), an individual’s willingness or openness to such valuable learning opportunities governs his exposure to TEK.

The same holds for prior entrepreneurial exposure (PEX). PEX is understood as an individual’s personal history related to entrepreneurship, such as entrepreneurial parents or prior work experience in entrepreneurial firms (Krueger, 1993; Zapkau et al., 2017). While the individual might not be an entrepreneur (yet), entrepreneurial insights are already being gathered through exposure from within the family or beyond, forming a breeding ground for possible future entrepreneurial development. This is how even nascent entrepreneurs tend to amass a certain amount of TEK. It has been argued that PEX facilitates the transfer of tacit knowledge, yet evidence remains sparse (Dohse and Walter, 2012). The introduction of TEK into the PEX-literature has the potential to shed light on this subject.

### Consequences of Tacit Entrepreneurial Knowledge

An individual who has amassed TEK has a distinct perspective on the entrepreneurial process and guides his venture based on personal insights and experiences (Hambrick, 2007). The idiosyncratic combination of experiences makes this knowledge valuable, rare, non-replaceable, and non-imitable (Barney, 1991), and therefore an asset to both the entrepreneur and the organization (Hambrick and Mason, 1984). This is reflected in a dichotomous disaggregation of the consequences of TEK. On the one hand, the consequences of TEK for the individual entrepreneur and, on the other hand, the consequences of TEK for the organization.

At the individual level, TEK can be considered an explanatory factor for nascent entrepreneurship. The impact of TEK on entrepreneurial intentions would be a fruitful research avenue to investigate since these entrepreneurial intentions are partly based on intuition or hunches (Bird, 1988), and the cognitive processes behind intentions are not yet fully understood (Krueger et al., 2000). As Ajzen’s (1991) theory of planned behavior is often used to study the entrepreneurial intentions of (nascent) entrepreneurs, it would be interesting to investigate the extent to which TEK influences entrepreneurial intent drivers. For example, TEK may serve as the antecedent of personal attitude, subjective norms, and perceived behavioral control in the decision-making process of becoming an entrepreneur.

Research on passion proliferates in entrepreneurship literature, mainly because of its positive impact on several vital outcomes (Murnieks et al., 2020). Yet to date, there is little understanding of the determinants of passion (Shepherd, 2015). Prior research has already identified several important stimuli for entrepreneurial passion, such as an individual’s social capital (Hoang and Gimeno, 2010), making way for TEK as an additional explanatory factor. TEK can be a possible impetus for this research, for instance, by investigating the relationship between an individual’s TEK and entrepreneurial passion (i.e., passion for inventing, founding, and developing) (Cardon et al., 2013). We contend that a high TEK-score, which equates to being proficient in entrepreneurship, results in a higher intense positive feeling associated with the different dimensions of entrepreneurial passion.

Finally, the prospect of inspiring and motivating followers is an essential prerequisite for many entrepreneurial founders. Although research on leadership in entrepreneurship is burgeoning, cognitive processes influencing a founder’s leadership behavior are sparse (Leitch and Volery, 2017). Jensen and Luthans (2006) emphasize the importance of prior experience in exhibiting leadership behavior. Consequently, TEK may also be applied as a potential explanatory factor for various leadership behaviors, such as transactional and transformational leadership.

At the organizational level, a crucial factor in the longevity of a venture’s success is its dynamic capabilities (Teece et al., 1997). A firm’s dynamic capabilities are defined as “the capacity (1) to sense and shape opportunities and threats, (2) to seize opportunities, and (3) to maintain competitiveness through enhancing, combining, protecting, and, when necessary, reconfiguring the business enterprise’s intangible and tangible assets” (Teece, 2007, p. 1,319). Organizational dynamic capabilities are closely intertwined with its founder, particularly in the context of a new business venture (Adner and Helfat, 2003; Teece, 2012). Both Zahra et al. (2006) and Teece (2007) have argued that entrepreneurial activities play a crucial role in the theory of dynamic capabilities since “the invisible hand must have fingers that can work in a coordinated fashion” (Augier and Teece, 2009, p. 410). Experiential learning lies at the heart of developing dynamic capabilities (Zollo and Winter, 2002), and this type of learning results from the tacit accumulation of experiences. Therefore, we believe TEK to be a driver for organizational dynamic capabilities. Moreover, we suspect founders who have amassed high TEK levels to influence their venture’s dynamic capabilities positively.

An additional role for TEK at the organizational level is evident in new ventures. New ventures are often initiated and led by a collective (Beckman, 2006; Schjoedt et al., 2013; Klotz et al., 2014), resulting in the convergence of people with different backgrounds, experiences, and mental models (Beckman, 2006; Leung et al., 2013). These emerging ventures depend on and utilize their new venture team members’ knowledge due to the absence of capital, organizational structures, and formalized rules (Gilbert et al., 2006; Dai et al., 2016). It is the new venture team’s collective perspective that steers organizational decisions (Mitchell et al., 2007; West, 2007). Many scholars find consensus for the fact that collective knowledge exceeds an individual’s cognitive capabilities (Knockaert et al., 2011; Bryant, 2014), but what knowledge is addressed or exchanged to elevate the collective remains unclear (de Mol et al., 2015). Moreover, how is valuable information regarding opportunities distributed among the collective? TEK represents a possible explanatory factor for the distribution of collective knowledge, considering that antecedents have received little attention in scientific research (Seyb et al., 2019).

### Contingencies

The relationship between internal characteristics and organizational effectiveness is highly influenced by contextual factors (Donaldson, 2001). We posit that this is also the case for an individual’s TEK. Certain contingencies can potentially influence the main effect between TEK and its antecedents or consequences. Since the individual is at the center of our model, several important contextual factors can be considered, such as firm size, industry, and institutional conditions.

Small and large companies are equipped with different resources and capabilities to interact with their business environment (Dean et al., 1998). The size differences are also apparent in the firm’s tacit knowledge resources (Antonelli and Scellato, 2015). Examples include a nascent entrepreneur who relies only on his own knowledge stock (Chrisman, 1999) or his new venture team (Schjoedt et al., 2013) to pursue his entrepreneurial endeavors. Large firms rely more on highly qualified personnel to innovate through formal R&D activities leading to intellectual property and patents (Antonelli and Scellato, 2015). Therefore, we expect a variance of firm size (either in prior or present work experience) to affect the central relation between TEK and its antecedents or consequences.

Much of the current literature on intra-firm knowledge transfer pays particular attention to the role of the industry. However, there are conflicting rationales for this role. On the one hand, some industries require a particular shared team background and experience to facilitate tacit knowledge transfer (Knockaert et al., 2011). On the other hand, there are industries where team heterogeneity is necessary to innovate and respond appropriately to external events or opportunities (Beckman, 2006). Given the differences in knowledge intensity, an exploration of the sector heterogeneity as a contingency variable on the process of accumulation and distribution of an individual’s TEK, in line with Park et al. (2015), would be an exciting research avenue.

The macro-economic perspective of entrepreneurship mainly explores a growth relationship, making way for a more deliberate focus on the individual’s unique sets of resources (Terjesen et al., 2016). Therefore, we believe that the introduction of TEK into the macro-economic perspective would unlock a broader perspective for the field. Scientific evidence suggests that institutional conditions, such as a nation’s start-up procedures, unemployment rate, income disparity, FDI inflows, and labor market conditions, can potentially impact a country’s total level of entrepreneurial activity (Casson and Wadeson, 2007; Chowdhury et al., 2015). Therefore, we posit that beneficial institutional conditions can enhance the relationships between TEK and its antecedent and consequences, respectively.

### Implications for Policy and Practice

This research has implications for different stakeholders in the entrepreneurial ecosystem for a variety of reasons. The TEK measure developed in this paper can be used by different stakeholders to reduce the information asymmetry related to the tacit knowledge of an entrepreneur. For instance, TEK can be a valuable instrument for banks to make an initial, preliminary assessment of entrepreneurs who turn up for a loan. Consistent with this argument, business angels and venture capitalists can make a better ex-ante assessment of new ventures and their entrepreneurs. In line with this reasoning, accelerators can integrate TEK into their selection processes. Moreover, the TEK measure developed in this paper can be used by entrepreneurs themselves in their search for new employees or cofounders. Namely, research suggests that companies need entrepreneurs *within* their organization (i.e., intrapreneurs) to drive innovation and explore new opportunities (Kraus et al., 2019). The TEK tool can help identify the tacit knowledge related to entrepreneurship of potential employees or cofounders during the recruitment process.

## Conclusion

Prior research and theory have suggested that a firm’s tacit knowledge is a crucial ingredient for success. However, tacit knowledge has many hidden secrets still to be discovered, especially its exploration on the individual level. Due to the longstanding empirical standstill, many research paths have remained unexplored. The introduction of an SJT as a valuable analytical tool makes way for a new stream of research to unravel the role of individual Tacit Entrepreneurial Knowledge (TEK). With the development of TEK, it is now possible to quantify an entrepreneur’s insights, experiences, and individual learnings (Sternberg et al., 2000) and its subsequent effect on organizational knowledge (Nelson and Winter, 1982; Kogut and Zander, 1992; Nonaka, 1994; Grant, 1996b). This article has endeavored to outline essential research gaps and promising research avenues for further research. We hope to revitalize inquiry on tacit knowledge in entrepreneurship and, by extension, in the broader management domain.

## Data Availability Statement

The raw data supporting the conclusions of this article will be made available by the authors upon request, without undue reservation.

## Ethics Statement

This study involving human participants was reviewed and approved by Prof. Dr. Liesbeth Todts and UHasselt Ethics Committee. The participants provided their written informed consent to participate in this study.

## Author Contributions

All authors listed have made a substantial, direct, and intellectual contribution to the work, and approved it for publication.

## Conflict of Interest

The authors declare that the research was conducted in the absence of any commercial or financial relationships that could be construed as a potential conflict of interest.

## Publisher’s Note

All claims expressed in this article are solely those of the authors and do not necessarily represent those of their affiliated organizations, or those of the publisher, the editors and the reviewers. Any product that may be evaluated in this article, or claim that may be made by its manufacturer, is not guaranteed or endorsed by the publisher.

## Footnotes

1 The complete TEK instrument is available from the corresponding author upon request.

2 An Excel form for the calculations is available from the corresponding author upon request.

3 348.755 is calculated by multiplying the critical value (i.e., 20.515) by the number of scenarios (i.e., 17).

4 None of the authors collaborating in this research study were included. The internal research team consisted of faculty members closely familiar with the concept of tacit knowledge (1 postdoctoral researcher, 1 associate professor and 1 full professor).
